# Intercellular Vesicular Transfer by Exosomes, Microparticles and Oncosomes - Implications for Cancer Biology and Treatments

**DOI:** 10.3389/fonc.2019.00125

**Published:** 2019-03-06

**Authors:** Ritu Jaiswal, Lisa M. Sedger

**Affiliations:** ^1^Faculty of Science, School of Life Sciences, University of Technology Sydney, Sydney, NSW, Australia; ^2^Discipline of Pharmacy, Graduate School of Health, University of Technology Sydney, Sydney, NSW, Australia

**Keywords:** cancer, cancer immunosuppression, cancer vaccine, exosome, extracellular vesicles, microparticle, pre-metastatic niche

## Abstract

Intercellular communication is a normal feature of most physiological interactions between cells in healthy organisms. While cells communicate directly through intimate physiology contact, other mechanisms of communication exist, such as through the influence of soluble mediators such as growth factors, cytokines and chemokines. There is, however, yet another mechanism of intercellular communication that permits the exchange of information between cells through extracellular vesicles (EVs). EVs are microscopic (50 nm−10 μM) phospholipid bilayer enclosed entities produced by virtually all eukaryotic cells. EVs are abundant in the intracellular space and are present at a cells' normal microenvironment. Irrespective of the EV “donor” cell type, or the mechanism of EV biogenesis and production, or the size and EV composition, cancer cells have the potential to utilize EVs in a manner that enhances their survival. For example, cancer cell EV overproduction confers benefits to tumor growth, and tumor metastasis, compared with neighboring healthy cells. Herein, we summarize the current status of knowledge on different populations of EVs. We review the situations that regulate EV release, and the factors that instruct differential packaging or sorting of EV content. We then highlight the functions of cancer-cell derived EVs as they impact on cancer outcomes, promoting tumor progression, metastases, and the mechanisms by which they facilitate the creation of a pre-metastatic niche. The review finishes by focusing on the beneficial (and challenging) features of tumor-derived EVs that can be adapted and utilized for cancer treatments, including those already being investigated in human clinical trials.

## Introduction

Several mechanisms of cell-to-cell and cell-to-microenvironment communication are used to maintain physiological processes in healthy organisms. These processes are numerous and often involve the production of soluble molecules such as cytokines and growth factors (1, 2). There is also an intercellular communication mechanism involving extracellular vesicles (EVs) (3). EVs are ubiquitous, but unlike soluble cytokines and growth factor molecules, EVs function as a vehicular-mediated exchange of surface and/or intracellular contents, delivering proteins, lipids, nucleic-acid based molecules and metabolites, between adjacent or distant cells, including within a tumor microenvironment (4, 5). In this review we discuss EV biology, the mechanisms of intercellular horizontal vesicular transfer and the impacts of EVs in cancer biology and cancer treatments.

EVs are microscopic phospholipid bilayer enclosed spherical bodies of approximately 50 nm – 10 μm in size. They are abundant particles that are often present in culture supernatants (*in vitro*) or present within tissue extracellular space (*in vivo*) between cells (6). The term EVs generally represents all kinds of vesicles released from any cell type, i.e. irrespective of the “donor” producer cell type, the biogenesis mechanism, the particle size, its composition or cargo. EVs are produced in normal cell physiology as well as in many pathological conditions. With respect to cancer, however, EVs play a role in tumor pathogenesis, starting from cancer initiation, propagation, formation of a pre-metastatic niche, and tumor migration, invasion and in cancer metastasis (7–9). As recent research has enabled a more in-depth understanding of the biology of EVs in cancer, it has become evident that EVs offer significant diagnostic and therapeutic potential. For example, cancer cell derived EVs can be used as a cancer biomarker(s) or to monitor the efficacy of cancer treatments (10). EVs are even being harnessed for cell-targeted drug delivery. Indeed, EVs are already being utilized in innovative biomedical and biotechnological applications including regenerative medicine, and tissue engineering, where they are being exploited for targeted drug delivery (11–13). So too, many other novel uses of EVs in cancer are being developed where they are being used for cancer drug monitoring or used as a cancer vaccine (14). Here we review the biology of EVs with respect to cancer and cancer treatments.

## Extracellular Vesicles: Exosomes, Microvesicles, Oncosomes and More

There is no unanimous consensus on the nomenclature of EVs largely because they are heterogeneous in nature. Generic terms such as “exosomes” and “microvesicles” have been broadly used, with different definitions depending on the context of the study. For example, descriptors such as tolerosomes (15), prostasomes (16), epididymosomes (17), etc., have been used to reflect tissue origin or a specific EV function (6). Here we will adhere, as much as possible, to the traditional nomenclatures of extracellular vesicles (EVs): microparticles (MPs) (or microvesicles, MVs) and exosomes.

**Figure 1:**
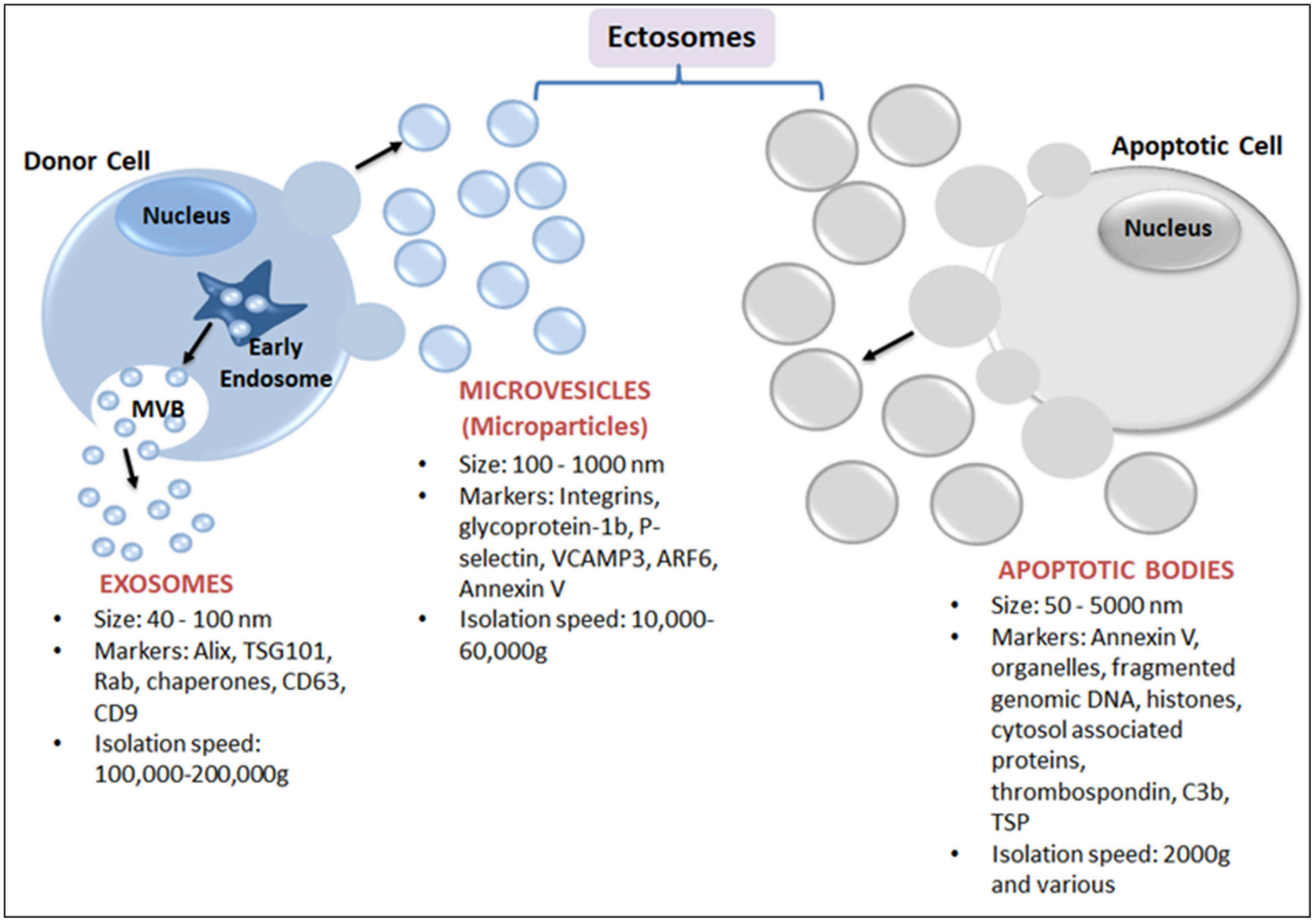
Schematic representation of major subtypes of EVs: exosomes, microparticles and apoptotic bodies. Exosomes, the smallest EVs, originate from within a cell by fusion followed by exocytosis of multivesicular bodies (MVB) from the cell membrane into the extracellular space. MVB are formed by the accumulation of luminal vesicles within endosomes. Ectosomes are assembled at, and pinched off from, the plasma membrane by a process of budding. Ectosomes include microvesicles (or MPs) released from activated cells and/or apoptotic bodies (produced from dying cells).

EVs are best defined based on their physical nature, size and biogenesis origin (Figure 1). Nevertheless, due to the biogenesis mechanisms, EVs are classified as either endosomes or ectosomes. The endosome as an organelle comprises internal membranes within the mammalian cell that ultimately fuses with the cells' plasma membrane, forming multi-vesicular bodies (MVB). These are categorized as intraluminal vesicles (ILVs) when present in the cytoplasm, or as exosomes when released into the extracellular milieu. Endosomal vesicles typically range between 40 and 100 nm in diameter (18), whereas ectosomes are shed directly by blebbing and budding mechanisms from the plasma membrane, and are considerably smaller, ranging from 100 nm to 10 μm (Figure 1). Ectosomes have also been referred to as microvesicles (MVs), microparticles (MPs), oncosomes, shedding vesicles, exosome-like vesicles or nanoparticles. Ectosomes include apoptotic bodies that are released from dying cells by blebbing and fragmentation of cell membranes; apoptotic bodies are typically 50–5000 nm in size.

EV terminology is sometimes reflective of EV cargoes. “Oncosomes” are 100–400 nm vesicles carrying abnormal and transforming macromolecules such as oncogenic proteins (19, 20). In other cases, EVs are known as large oncosomes (LO) since they are distinct from other EVs and typically 1–10 μm in size (21). LOs can be produced from tumor tissues including human prostate cancer (22) and breast cancer (23). However, naming EVs according to their tissue of origin raises confusion because both malignant and non-malignant cells can produce EVs. For example, prostate epithelial cells release EVs that are present in semen (16, 24) and although these EVs are sometimes referred to as “prostasomes,” they are either exosomes or microvesicles—depending on their endocytic or plasma membrane origin (16). Moreover, the term prostasome broadly refers to *all* EV-like particles that are present in semen plasma i.e., EVs produced from *any* male urogenital cell type (16, 24). Indeed, the tendency of naming EVs based simply on the biological fluid from which they were isolated has resulted in a somewhat confusing set descriptive terms such as epididymosomes, migrasomes, promininosomes, vexosomes, dexosomes, cardiosomes, texosomes etc. (17, 25, 26). It is important to realize that these terms show no relationship to EV biogenesis or EV functions.

EV-like particles can also be produced from virus-infected cells, such as Herpes virus and retrovirus infected cells. These EVs are typically produced from the host cell plasma membrane and they contain viral-gene encoded molecules (27, 28) but generally lack viral genomes, making them non-infective (29) - for review see (30). Additionally, Golgi organelle membrane-derived EVs known as “gesicles” are released from vesicular stomatitis virus (VSV) DNA transfected cells. These EVs contain the VSV glycoprotein that confers fusogenicity (31, 32) and have a lower density relative to conventional exosomes (33). Nevertheless, non-infected cells can also produce Golgi vesicle derived EVs that are present in body fluids, contain Golgi and endoplasmic reticulum (ER) proteins, and are packaged and secreted as transport vesicles (34). The extent to which virus-induced oncogenesis influences EV production, for example, in HPV-induced head and neck cancer, or HPV-induced cervical cancer, is still unknown and this requires significant further investigation.

## Sources of Extracellular Vesicles

EVs are secreted constitutively or following cellular activation and are identifiable in *in vitro* cell culture supernatants and in *in vivo* biofluids. EVs can be produced by virtually any mammalian cell type - irrespective of the health status of the cell. EVs are present within blood (35) [plasma (36)], semen (37), urine (38) saliva (39), sputum (40), breast milk (41), amniotic fluid (42), ascites fluid (43), cerebrospinal fluid (44), bile (45), bronchoalveolar fluid (46), malignant ascites (47), lymphatic fluid (48), nasal secretions (49), in tears (50), and are even abundant in feces (51). EVs in body fluids reflect the normal biochemical and metabolic processes of their origin cells. However, EVs may or may not primarily be representative of the most predominant cell type within a specific tissue. For example, EVs in blood have properties of blood vessel endothelial cells, or of the cellular components of the blood itself such as leukocytes, erythrocytes or platelets and the relative abundance of each of these EVs can change depending on the physiological situation (52). In humans EVs are often most abundant in biological fluids that are released externally, such as breast milk, saliva and urine, and they are less abundant in non-secretory type fluids i.e. physically enclosed or contained fluids such as blood and cerebrospinal fluid (53). The fact that EVs are molecularly reflective of their tissue of origin is particularly significant in the context of cancer because tumor cell derived EVs contain molecules that are often specific to their neoplastic origin. For example, exosomes in the blood of brain tumor patients contain more neural cell adhesion molecules and brain tumor antigen L1NCAM (CD171) relative to EVs in blood of healthy individuals (54). In other examples, exosomes from melanoma patients contain Melan-A/Mart1 (55), and EVs in urine from urogenital cancer patients can contain elevated CD36, CD44, 5T4, basigin, CD73, which are all markers of specific malignancies (56–59).

## Modulation of EV Production

EV production and release can be altered and regulated; EV production can be triggered by internal cellular process or external stimuli. On the other hand, normal EV production can also be suppressed. Interestingly, there is evidence that cancer cells produce greater numbers of EVs compared to non-transformed healthy cells (60–62) and the likely stimuli for this phenomenon are many. For example, EV production can be enhanced by chemotherapy or photo-dynamic treatments, and sometimes this contributes to the disease burden of the patient (63). Interestingly, a single cell type can produce several types of EVs, as shown for platelets (64), endothelial cells (65) and breast cancer cells (66) that produce both exosomes and MVs. Furthermore, different stimuli can change the production of EV types and vary cargo levels, or even post-transcriptional cargo modifications (67, 68). Thus, factors such as the stimuli that triggers EV release, the donor cell type, and its normal physiological or disease condition, or the biogenesis pathway(s), influence the characteristics and abundance of the EVs present in human biological fluids, especially in situations of pathology including cancer.

### Factors Stimulating EV Production

Multiple factors can influence EV shedding (see Table 1). The involvement of fusion machinery such as the SNARE (soluble NSF [*N*-ethylmaleimide-sensitive factor] attachment protein) receptor SNAP, and tethering factors, are indicated in stimulating EV biogenesis and release [reviewed in (98)]. However, factors such as temperature, cell membrane receptor activation status, infection, or stress, also contribute to the process. For example, lipopolysaccharide-stimulated dendritic cells, and antigen or mitogen activated B and T lymphocytes increase EV production *in vitro* (75, 99). This has implications in the setting of cancer immunotherapy where immune-modulating biologics are showing impressive efficacy in previously difficult-to-treat cancers, and where EVs are likely to be useful for cancer treatment monitoring. Cell stress is a particularly important factor in cancer because stress-induces intracellular calcium triggers and increased EV production from cancer cells (79). Interestingly, certain gene polymorphisms can correlate with increased EV production capability. Mechanisms vary but in the case of the A348T P2X_7_R polymorphism, this amino acid substitution results in ATP increased IL-1β secretion by monocytes, and IL-1α IL-1β and IL-18 which stimulates increased secretion of EVs (100, 101).

**Table 1:** Factors stimulating EV release.

**Mechanism of induction of EV release**	**EV size (nm)**	**EV source**	**EV content**	**References**
**EXOSOMES AND EXOSOME-LIKE VESICLES/EXTRACELLULAR VESICLES**				
• Tumor necrosis factor (TNF)	20–50	Human bronchial epithelial cells	TNF-R1, TRADD	(69)
• Inhibition of oncogenic Epidermal Growth Factor Receptor Kinase	30–100	Human cancer cell lines	EGFR, P-EGFR, & exo-gDNA	(70)
• Heparanase	30–120	Human myeloma cell line	Syndecan-1, VEGF & HGF	(71)
• Hypoxia	30–100	Human breast cancer lines	Elevated miR-210	(72)
• Plasma membrane depolarization	40–100	Neurones and astrocytes	Cell adhesion and membrane proteins	(73, 74)
• Cross-linking of CD3 • (TCR activation)	50–100	Jurkat T cells or T lymphoblasts	CD3/TCR, CD2, LFA-1, MHC-I and II, & CXCR4	(75)
• Glutamate	50–100	Oligodendrocytes in the brain	Cre-recombinase	(4)
• Induction of the oncogene Wnt5A	ND	Melanoma cell line	IL-6 & the pro-angiogenic factors IL-8, VEGF & MMP2	(76)
• Activation of Her2 by ligands EGF and Heregulin	ND	Her2 overexpressing breast cancer cells (BT-474)	Activated Her2	(77)
• GAIP interacting protein C • Terminus (GIPC) depletion	40–100	Pancreatic cancer cell line	Overexpression of drug resistance gene ABCG2	(78)
**EXOSOMES AND MICROVESICLES (MVs)**				
• Increasing intracellular Ca^2+^ by: • Thrombin receptor activation via • α-thrombin or thrombin-receptor • Activating peptide (TRAP)	Exo: 40–100 MV:100–1000	Platelets from human whole blood	Exosomes: CD63 MV: Integrin & P-selectin	(64)
**EXOSOMES AND GIANT MULTIVESICULAR BODIES (MVB)**				
• Increased intracellular Ca^2+^: Monensin ionophore & activation of transferrin receptor	60–100	Human erythro-leukemia cell line	ND	(79, 80)
**INTRALUMENAL VESICLES**				
• Increasing intracellular Ca^2+^: Calcium containing media	60–80	Mice bone marrow-derived mast cells	MHC-II	(81)
**MVs AND APOPTOTIC VESICLES**				
• Increasing intracellular Ca^2+^: P2X7 activation via ATP	250–2000	Microglia	Pro-IL-1β	(82)
**MICROVESICLES (MVs)**				
• Increasing intracellular Ca^2+^: 1. P2X7-R activation by ATP	<0.5 μm	THP-1 monocytes	Bioactive IL-1β	(83)
2. ATP-mediated activation of P2X7R	ND	RAW MØ	Intracellular isoform of IL-1ra	(84)
3. Activation of PAK1/2 via Cdc42 & Rac1-dependent pathways by thrombin receptor-activating & collagen or calcium ionophore	<1.0–1.5 μm	Platelets	Cortactin, filamin A and actin	(85)
• Hypoxia and gamma radiation	<1 μm	MVs- Human & murine lung cancer cell lines	ND	(86)
• Elevated peptidylarginine deiminases (PAD2 & PAD4) induced by BzATP stimulation of P2X7 receptors	200 nm average	Prostate cancer cell line	ND	(87)
• EGF Treatment (activation of Rho & ROCK)	≤0.22 μm	Human cervical HeLa cells	ND	(88)
• Phorbol 12-myristate 13-acetate (PMA)	≤1 μm	Human cancer cell lines	HLA Class- I, CD29, CD44v7/8, CD51, chemokine receptors CCR6 & CX3CR1, extracellular matrix metalloproteinase inducer (EMMPRIN), epithelial cell adhesion molecule (EpCAM)	(89)
• Activation of P2X7 via ATP	0.5–1 μm	Macrophages	Phospholipids	(90)
• Respiratory Infections (LPS, live *H. influenzae* bacteria or viral mimetic Poly:IC)	20–1000 nm 100–400 nm	Broncho alveolar lavage fluid (BALF) from mice	ND	(91)
**MICROVESICLES (MVs) OR MICROPARTICLES (MPs)**				
Overexpression of v-H-RAS	ND	Melanoma cells	MMP-2	(92)
**MICROPARTICLES (MPs)**				
• Thrombin induced activation of Rho & ROCK-II pathway	<1 um	Human microvascular endothelial cell line	ND	(93)
• Combination of ionizing radiation & TNF (stimulation of ROS)	<1 um	Human umbilical vein endothelial cells	Tissue Factor (TF)	(94)
• Activation of acid A-SMase by benzoyl-ATP	100 nm−1 μm	Glial cells	A-SMase	(95)
**EXTRACELLULAR VESICLES (EVs)**				
• Sub-lethal photodynamic treatment, cytotoxic insult	300–400 nm	Human prostatic cancer cells *in vitro* and in *in vivo* in mouse	Apoptotic markers, drugs from their parent cells, tumor membrane & endosome contents	(63)
• Activation by LPS	0.1–5 μm	Dendritic cells	ND	(96)
**GESICLES**				
• Overexpression of VSVglycoprotein	100 nm	Human kidney and lung cell lines	VSV-G	(32)
**ONCOSOMES**				
• Activation of EGFR & AKT Pathways	0.5–5 μm	Prostate cancer cells	Caveolin-1	(20)
• Silencing of the cytoskeletal regulator diaphanous-related formin-3 (DIAPH3) by ERK	>1 μm	Prostate cancer cell line (DU145)	miR-125a	(97)

*ND, Not defined*.

### Factors Attenuating EV Production

EV release may be negatively modulated. This can be important in the context of EV-mediated disease pathogenesis since decreasing production may alleviate the extent of disease burden, e.g., in cancer patients. Drugs that block EV biogenesis, inhibit EV release, EV uptake (by recipient cells), or that interfering with EV-specific recipient cell signaling, are already known. Although the potential to regulate EV production for therapeutic benefit is still very much in its infancy, this approach appears to have particular relevance to cancer pathology, as well as to cancer detection, and cancer treatment outcomes—as is evident in the latter sections of this review.

#### Blocking EV Biogenesis

Effector molecules that are associated with the membrane vesicle formation or biogenesis processes may be targeted to inhibit the microvesiculation process. As expected intracellular vesicular transport is integral to EV biogenesis, as demonstrated in cells treated with dimethyl amiloride (a drug for clinically managing hypertension) that lowers the yield of tumor-derived EVs by interfering with the recycling of endocytic vesicles (102). This drug functionally abrogates EV-mediated immunosuppressive effects *in vivo* in a mouse tumor model (102). The lipid translocase enzyme systems are equally important. These include the lipid membrane flippases, scramblases and floppases that are involved in the process of plasma membrane processing to maintain phospholipid symmetry, cytoskeletal modeling and vesicle budding (5). The critical importance of scramblases and sheddases to EV production has been demonstrated via the reduction of MV shedding in human erythrocytes in the presence of the R5421 scramblase inhibitor (103), or the ADAM17 disintegrin metalloproteinase i.e., typical sheddase enzymes (104). Sphingomyelinase-2 plays an important role, too, as it controls the accumulation of ceramide—a physiological trigger for apoptotic cell membrane blebbing and budding of exosomes from multivesicular endosome membranes (105). Indeed, sphingomyelinase-2 inhibitor GW4869 influences exosome-mediated tumor growth, lowering the number of lung metastases in tumor bearing mice (106, 107). Similarly, neutral sphingomyelinase (N-SMase) contributes to exosome biogenesis as demonstrated in oligodendrocytes whereas acid sphingomyelinase (A-SMase) activity is required for MP release from microglial cells (95, 105). Consistent with these findings, an A-SMase inhibitor, imipramine, blocks microvesiculation processes in prostate cancer cells by preventing the activation and movement of A-SMase to the plasma membrane (108). Together these studies demonstrate that different SMase-family enzymes are specific for modulating the production and release of discrete populations of EVs.

Endocytosis inhibitors can influence EV production. Chlorpromazine and methyl-β-cyclodextrin impede the release of exosomes in human prostate cancer cells *in vitro* (108). The suppression of the RhoA/ROCK-dependent signaling pathway by a ROCK inhibitor Y-27632 reduces the secretion of MVs from human breast cancer cells, human primary glioblastoma cells and EGF-stimulated HeLa cells *in vitro* (88). RhoA and the highly-related GTPases Rac and Cdc42 are implicated in regulating MV shedding from transformed cell lines and controlling the packaging of specific cargo into MVs (109). Interestingly, the calcium-dependent activation of peptidylarginine deiminase enzymes are elevated in cancer (48) but this can be pharmacologically inhibited by Cl-amidine, which is a peptidylarginine deiminase inhibitor that reduces microvesiculation (108).

#### Blocking EV Release

Inhibition of vesicular release can be achieved *in vitro* by targeting many steps of vesicular body processing at the plasma membrane. For example, MV shedding is inhibited in various tumor cell lines (including human melanoma, colon cancer, prostate adenocarcinoma and breast tumor cell lines) where the GTP-binding protein ARF6 activation is inhibited; ARF6 regulates plasma membrane endosomal trafficking (66). *In vitro* experimental knock-down of Rab27a or Rab27b impairs exosomal secretion in Hela cells without effecting normal physiological protein secretion (110), and Rab11 and Rab35 GTPases prevent exosome release by compromising the integration of multi-vessicular bodies with the cells plasma membrane (111). Consistent with this, decreased endothelial cell MP release can be achieved by treatment with the Y27632 Rho-kinase inhibitor (112). Indeed, drugs such as bisindolylmaleimide-I (a protein kinase C inhibitor) prevent the release of EVs, by inhibiting the externalization of phosphatidylserine (113); the subsequent non-externalization results in the suppression of exosome and MV release from prostate cancer cells (108).

Coincident with the many successful examples of *in vitro* drug-induced suppression of EV production and/or release there are now numerous exciting clinical examples where this has also been achieved *in vivo*. The significance is both with respect to EVs in disease pathogenesis, and as biomarkers of cancer cure and/or relapse. For example, a decrease in platelet MV production has been reported with calpain inhibitors calpeptin (85), calpastatin, MDL 28, 170, E64d (114) or thiosulfinates (115). Pre-treatment of endothelial cells with anti-oxidants pyrrolidine dithiocarbamate and *N*-acetylcysteine reduces the release of thrombogenic tissue factor-bearing MPs, and decreases apoptosis and reactive oxygen species production (94). Other therapeutic targets include intracellular Ca^2+^ channels because channel inhibitors such as nifedipine and benidipine are effective in decreasing EV release (116), and, moreover, proton-pump inhibitors result in an acidic environment and thus inhibition of melanoma cell exosome release (117). Furthermore, vitamin C therapy inhibits MP plasma levels (118), and since a number of cytokines including interleukin-1β and tumor necrosis factor can induce EV release from specific cells, then, so too, suppression of cytokine synthesis or blocking cytokine receptor function can ultimately reduce EV production (95, 119).

Taken together there is a large body of data documenting a diverse spectrum of EV inhibitory agents, which indicates that certain drugs may tend to act on all types or categories of EVs. There are, however, some pharmacological molecules have been shown to impact the release of specific types of EVs. For example, cytochalasin D appears to decrease in exosome size whilst increasing the production in MV sized vesicles (108). The same study also demonstrated the inhibition of exosome release (but not MVs) by methyl-β-cyclodextrin, whereas Y27632 decreased MV release alone (not influencing exosome production/release). Because many proteins associated with the biogenesis and trafficking of EVs are important in normal functions, therapeutic inhibition of EV production may have unfavorable consequences or off-target effects *in vitro* and *in vivo*. Achieving a therapeutic inhibition of EV production is the focus of much research and several clinical trials (see Table 2).

**Table 2:** Summary of clinical trials using extracellular vesicles^a^.

**Vesicle type**	**Cancer type**	**Trial phase**	**Trial aim|(s)**	**Trial status**	**Application**	**Trial number**
**PART I: EXOSOMES**						
Plant exosome	Colon cancer	I	Investigating the ability of plant exosomes to deliver curcumin to normal & colon cancer tissue.	Active, not recruiting	Drug delivery	NCT01294072
Urinary exosome	Prostate cancer	**–**	Clinical validation of a urinary exosome gene signature in men presenting for suspicion of prostate cancer.	Completed	Biomarker	NCT02702856
Exosome	Malignant solid tumors	**–**	Quantify a stress protein in the blood and in the urine for the monitoring and early diagnosis of malignant solid tumors.	Recruiting	Diagnostic	NCT02662621
Exosome	Esophageal adenocarcinoma	**–**	Evaluation of MicroRNA expression in blood and cytology for detecting Barrett's esophagus & associated neoplasia.	Recruiting	Diagnostic	NCT02464930
Exosome	Ovarian cancer	**–**	To see if monocytes taken from the blood of people with ovarian cancer can kill tumor cells (exosomes, may influence outcome).	Completed	Mechanistic	NCT02063464
Urine exosome	Thyroid cancer	**–**	Anaplastic thyroid cancer & follicular thyroid cancer-derived exosomal analysis via treatment of lovastatin and vildagliptin & pilot prognostic study via urine exosomal.	Not yet recruiting	Biomarker	NCT02862470
Onco-exosomes	Pancreatic cancer	**–**	Diagnostic accuracy of circulating Tumor cells (CTCs) and onco-exosome quantification in the diagnosis of pancreatic cancer.	Recruiting	Diagnostic	NCT03032913
Exosome	Exosome	**–**	Circulating exosomes as potential prognostic and predictive biomarkers in advanced gastric cancer patients: A prospective observational study.	Unknown	Biomarker	NCT01779583
Exosome	Cholangiocarcinoma	**–**	Characterization of the ncRNAs in tumor derived exosomes from cholangiocarcinoma patients before anti-cancer therapies & benign biliary stricture patients.	Recruiting	Recruiting	NCT03102268
Exosome	Oropharyngeal squamous cell carcinoma	**–**	Exosome testing as a screening modality for human papillomavirus-positive oropharyngeal squamous cell carcinoma.	Recruiting	Screening biomarker	NCT02147418
Plant exosome	Head and neck cancer	**–**	Evaluation of the ability of edible plant exosome to prevent oral mucositis associated with chemo radiation treatment of head & neck cancer.	Recruiting	Drug delivery	NCT01668849
Exosome	Head and neck cancer	I	Studies how well metformin affects cytokines & exosomes in patients with head & neck cancer.	Recruiting	Drug effect	NCT03109873
Exosome	Non-small cell lung cancer	**–**	Consistency analysis of PD-L1 in cancer tissue & plasma.	Not yet recruiting	Diagnostic	NCT02890849
Exosome	Non-small cell lung cancer	**–**	Consistency analysis of PD-L1 in cancer tissue & plasma exosome.	Not yet recruiting	Diagnostic	NCT02869685
DC-derived exosomes	Non-small cell lung cancer	II	Consistency analysis of PD-L1 in lung cancer tissue and plasma exosome before & after radiotherapy.	Unknown	Vaccine	NCT01159288
Exosome	Metastatic melanoma	**–**	Study of molecular mechanisms implicated in the pathogenesis of melanoma. Role of exosomes.	Recruiting	Mechanistic	NCT02310451
Exosome	Lung metastases osteosarcoma	**–**	Whether the profile of RNA from circulating exosomes can be used as a biomarker for lung metastases of primary high-grade osteosarcoma.	Recruiting	Biomarker	NCT03108677
Exosome	Pancreatic cancer	**–**	Interrogation of exosome-mediated intercellular signaling in patients with pancreatic cancer.	Recruiting	Mechanistic	NCT02393703
**PART II: MICROPARTICLES**						
Microparticles	Breast cancer	II	Assess the reduction of tissue factor bearing microparticles in metastatic breast cancer treated with rosuvastatin.	Active, not recruiting	Treatment	NCT01299038
Microparticles	Advanced pancreatic, colon, lung, gastric and ovarian		The cumulative incidence of VTE at 2 months in the higher venous thrombo-embolic events in cancer patients with high levels of circulating tissue factor bearing microparticles (TFMP).	Completed	Diagnostic	NCT00908960
Microparticles	Deep vein thrombosis (DVT) and cancer		Determine the prevalence of asymptomatic lower extremity DVT detected by US-doppler and pro-coagulant microparticles in a selected group of cancer patients suffering from an advanced stage of the disease.	Completed	Diagnostic	NCT00336258
Microparticles	Myeloproliferative neoplasm (MPN)		Platelet microparticles are involved in the hypercoagulability of MPNs patients.	Completed	Mechanistic	NCT02862366
Microparticles	Cancer, deep venous thrombosis, pulmonary embolism		To identify cancer patients at high risk for VTE based on clinical characteristics, coagulation biomarkers & the coagulant activity of tissue factor bearing microparticles.	Completed	Biomarker	NCT02095925
Microparticles	Malignant pleural effusion	II	To investigate the anticancer effect and the related immunological mechanism of methotrexate-autologous tumor derived microparticles (MTX-ATMPs) in the treatment of malignant pleural effusion.	Recruiting	Drug delivery	NCT02657460
Microparticles	Venous thromboembolism, pulmonary thromboembolisms, cancer		Carrying out a study in cancer-associated-thromboembolism patients in order to decide the suitable anticoagulation time. Pro-coagulant role of phospholipid-dependent microparticles.	Completed	Diagnostic	NCT03134820
Tumor-derived microparticles	Malignant pleural effusion, malignant ascites	II	Safety and effectiveness study of tumor cell-derived microparticles to treat malignant ascites and pleural effusion.	Unknown	Treatment	NCT01854866
Microparticles	Colon cancer	**–**	Examining the relationship between relaxation combined with biofeedback or wheat germ juice to the immune indices and quality of life measures in patients with colorectal cancer who receive prophylactic chemotherapy after surgery.	Recruiting	Mechanistic	NCT01991080
Tumor-derived microparticles	Pancreatic cancer	III	Safety and efficacy of clopidogrel in loc-ally advanced & metastatic pancreatic adenocarcinoma treated with chemotherapy.	Recruiting	Drug safety & efficacy	NCT02404363
Microparticles	Hepatic, pancreatic and colorectal neoplasms	**–**	To investigate quantitative and qualitative aspects of microparticles during cardiac and abdominal operations.	Completed		NCT00677781
Microparticles	Acute lymphoblastic leukemia	**–**	Role of the microparticles and of tissue factor in the pro-thrombotic phenotype and the thromboembolic complications during the acute lymphoblastic leukemia in children.	Completed	Diagnostic	NCT02862652
Microparticles	Prostate cancer		Evaluation of a novel circulating microvesicle-based multi-analyte assay for the detection of prostate cancer in men with elevated risk for prostate cancer.	Completed	Diagnostic	NT01499381

a *Retrieved from https:clinicaltrials.gov/ct2/search as on 17/10/2018*.

## Biochemical Features of Extracellular Vesicles

EVs package many bioactive materials such as nucleic acids comprising of DNA and RNA molecules—including coding RNAs (e.g., messenger RNAs), as well as non-coding RNAs e.g., long non-coding RNAs (lncRNAs), microRNAs (miRNAs) and circular RNAs (120). Transmembrane surface proteins e.g., signal-transducing receptors, or intracellular proteins including a range of cytoplasmic transcriptional factors have all been demonstrated to be present in EVs. Consistent with this, even carbohydrates and glycans, and lipid-based molecules, are packaged in EVs, along with biochemical metabolites (121).

The diverse range of EV cargo is well documented in online listings: *ExoCarta* (www.exocarta.org) (122) and *Vesiclepedia* (www.microvesicles.org) (123). *Vesiclepedia* currently lists 349,988 proteins, 27,646 mRNA, 10,520 miRNAs and 639 lipids, spanning 41 species (both animals and plants) from approximately 1254 independent studies (database accessed on January 2019). A review of this register reveals that certain proteins appear to more frequently packaged and transported by certain types of EVs. Furthermore, the EV registers enable the identification of specific molecules as markers of the different types of EV. For instance, exosome biomarker proteins comprise ESCRT proteins Alix, TSG101, CD9, CD63, CD81, chaperones HSC70 and HSP90, ceramide, flotillin, Rab, and tetraspanin family members (67, 105, 124, 125), and MVs, owing to their plasma membrane origin, appear to predominantly contain integrins, glycoprotein-Ib, P-selectin, VCAMP3, and ARF6 (27, 64, 126). MVs are typically characterized by the presence of externalized phosphatidyl serine—like apoptotic bodies (127). (Of note: EVs are distinguishable from MPS because, apoptotic bodies contain fragmented genomic DNA and even histones,—unlike the exosomes or MPs—and additional phenotypic markers of apoptotic bodies are thrombospondin, and complement component C3b (27, 128–130).

In the same manner certain proteins and miRNAs can identify LO's, including cytokeratin 18 (in prostate cancer derived LOs) or the miR-1227 (in prostrate epithelial cell LOs), compared to smaller sized EVs (21, 131). Of note: LO cargoes are functional and fully capable of inducing an EV-recipient cell effect, just like other EVs (131). However, various “EV type” markers are not exclusive and considerable overlap exists. This is attributable to the heterogeneity in the EV populations, and/or to imperfect EV isolation procedures.

## Packaging and Sorting of EV Content

The compartment of origin of EVs, and the EV cargo, are both influential to the types of intercellular interaction and information that is delivered to a recipient cell. For example, MVB-derived exosomes (compared to plasma membrane derived MVs) differ in their cargo content (66, 132, 133). This sorting or selective content packaging is regulated at multiple levels. The endosomal sorting complex required for transport (ESCRT)-dependent pathway is involved in the selection and distribution of proteins within exosomes (134). CD63-dependent process may also be involved in sorting EV cargo (135) and ARF6-regulated recycling pathways affect the packaging of major histocompatibility class I (MHC-I) molecules, integrin receptors, vesicle associated protein-3 and membrane matrix metalloproteinases, whereas ARF6 directs cargo selection in MVs (66). The sorting of nucleic acids in EVs is less well understood but ribonucleoproteins are involved in RNA molecule sorting (136–138). So, too, the RNA-induced silencing complex (or RISC) (136) and heterogeneous nuclear ribonucleoprotein hnRNPA2B1, as these load miRNA into EVs (137), while the Y-box protein-1 aides encasing of miRNA into exosomes (138). The presence of the RNA biogenesis machinery in EVs, suggests that miRNA biogenesis can occur within the EV, which provides additional significance as this offers the capacity for altering recipient cell gene expression (i.e., by newly produced miRNA within the recipient cell) (139).

Since the protein and RNA content of EVs closely matches the donor cell, thus the presence and absence of such molecules offer a snapshot of the molecular circumstances of the donor cell—*precisely* at the time when the EV is produced. However, as noted earlier, certain conditions or factors, such as hypoxia, heat stress, oxidative stress (140, 141), infection (142) or cell activation (143) [including the activation of specific signaling pathways (144)] result in alterations in EV cargos. Indeed, hypoxia induces alterations of both protein and RNA from endothelial (145) and tumor cell-derived exosomes (72). Also, exosomes from human glioblastoma multiforme patients (serum and tumor samples), or primary glioblastoma cell lines, are elevated in VEGF2 and EGRFA2, in response to hypoxia, and this induces endothelial cell sprouting (*in vitro*)—an indication of *in vivo* angiogenesis potential (146). Furthermore, stress and apoptosis induce modulation of mRNA content molecules in exosomes (141), particularly heat-shock proteins (HSPs) (140). In other cancer-related examples, the expression of oncogenes such as mutant KRAS (147) or HRAS (148) modulates the composition of exosomes, and cytotoxic chemotherapy induces expression of phosphatidyl serine and tissue factor in MPs (149), and ERBB2/Her2 oncogene overexpression in EVs mediates transformation toward a malignant phenotype (150). Of note, on a gram per gram basis many comparisons of donor cells and their EVs show differential expression of certain molecules, suggestive of molecule enrichment via a *selective packaging* mechanism. Knowledge of why the EVs might contain more or less of a specific molecule (relative to its donor cells) is still incomplete. There may, however, be benefits or disadvantages to the host organism, or these differences may merely be reflective of the membrane: cytoplasm ratio and the inherent properties of each molecule.

## EV Target Cell Interactions

EVs have the capacity to interact with a wide variety of recipient cells i.e., any cell that engages with EVs, be it within tissue extracellular space, during or after blood or lymphatic distribution. This means that either the original EV donor tissue cells, or blood or lymphatic endothelium, or a distant tissue cell can be influenced by EVs. EVs therefore serve as an important vector of intercellular molecular exchange in diverse but both physiologically linked and unrelated tissues.

### EVs as Carriers of Nucleic Acids or Proteomic Molecules

It has long been known that EVs are carriers of genetic and proteomic information. It has not, however, always been appreciated that EV trafficked molecules can be present in a relatively concentrated form. In this regard EVs are frequently said to be “rich” in expression of certain molecules, for example, we and others have shown, EVs can contain high concentrations of certain molecules, such as p-glycoprotein - relative to the EV donor cells (151) Examples of this enrichment phenomenon include proteins, miRNAs and even metabolites (58, 121, 152, 153) and this engenders EVs with genuine capacity to confer functional effects on recipient cells.

The EV cargos influence the recipient cells in multiple positive ways; in normal circumstances EVs are important in tissue homeostasis and organogenesis (52). For example, platelet derived microvesicles induce angiogenesis *in vivo* (in mice) by facilitating the formation of endothelial capillaries (154) (by virtue of their cytokines-VEGF, bFGF, and PDGF cargo). In pathological situations, however, certain EV functions are implicated in contributing to acute and chronic diseases. In malignancies, the released EVs propagate cancer-signaling molecules such as oncoproteins including epidermal growth factor receptor-III (EGFRvIII), mutant Ras family members, or c-Met etc. (19, 148, 155). Oncogenic transcripts potentially contribute to the horizontal transformation mechanisms and phenotypic reprogramming of the recipient cells (156). Clinically they can serve as cancer biomarker(s) i.e., tumor presence, or as an indication of cure or remission (21, 157). They can also be indicative of cancer staging at the time of diagnosis (158).

### EV-Recipient Cell Uptake

The function(s) of EVs depends on their ability to interact with a recipient cell in a manner that inherently involves direct contact of membranes. When encountering a recipient cell, EVs may simply adhere and stably associate with the recipient cell membrane surface, then either dissociate, or become incorporated within the recipient cell membrane (159). Recipient cell EV internalization occurs via multiple mechanisms. Both passive processes of membrane fusion (160), and active processes involving clathrin- and caveolin-dependent internalization, are used in EV-recipient cell interactions and the process typically involves membrane lipid rafts and endocytosis (161–163). EV internalization can also occur by phagocytosis (164), micropinocytosis (165), or macropinocytosis (166). The EV:recipient cell interaction can additionally involve specific ligand-receptor type interactions, such as between membrane-bound cytokines and their cytokine-specific receptors, including TNF-family molecules TNF, TRAIL or FasL and their receptors (167). The EV:recipient cell interaction is therefore both generic and specific. Furthermore, the involvement of transmembrane proteins explains the capacity of EVs to act as activators of specific intracellular signaling pathways (168) that can can be highly specific, even for receptor isoforms and downstream signaling events, as has been shown for exosome VEGF in activating VEGFR (159).

It is unclear whether EV internalization is selective or a completely random process. Current evidence suggests a degree of cellular control of EV internalization, dependent on factors such as the target cell type, the location, or physiological and environmental conditions, or even dependent even on the molecule being received/internalized e.g., nucleic acid, protein, carbohydrate, lipid. In most cases, however, the recipient cell specific interactions with EVs are regulated by adhesion molecules (161). For example, EV phosphatidyl serine, tetraspanin (169), ICAM-1, galactin-5 and galectin-9 (170), TIM4 phosphatidyl serine-specific receptors (164, 171) and heparan sulfate proteoglycans, on the recipient cell surface are involved in the internalization of EVs (172). Additionally, EVs expressing syncytin 2 (also present on a number of tumor cells) (173) bind to a recipient cell specific receptor MFSD2a (Major Facilitator Superfamily Domain 2a), permitting fusion of the EV:recipient cell membranes (168). Fusion permits the exchange of transmembrane proteins such as transfer major-histocompatibility class-II molecules, which, with respect to cancer, will transiently change the antigenicity (antigen presentation capacity) of the recipient cell (170) (also see section Current Challenges in the Use of EVs in Oncology). It has become clear that the number of EV transmembrane molecules engage with, and are exchanged with, recipient cells, and the temperature at which these interactions occurs contributes to the efficiency of EV uptake and the types of molecular membrane exchanges (165). The significance of these interactions is that the release of EV cargo into the recipient cells' cytoplasm can confer a new functional capacity in the recipient cell. This does not necessarily complete the intercellular communication process as the transferred cargo may accumulate into recipient cell vesicles that are subsequently released i.e. liberated to fuse with yet another target cell. Again, in the context of cancer, the EV-mediated intercellular dissemination of bioactive oncogene proteins from one cell type to other cell may in turn contribute to the development of secondary tumors at distant sites (155, 174). This can occur even though the germline DNA mutation of the oncoprotein is not germline encoded within the recipient cell *per se*.

## Biological Effects of Tumor-Derived EVs in Cancer Progression, Migration and Oncogenic Survival

**Figure 2:**
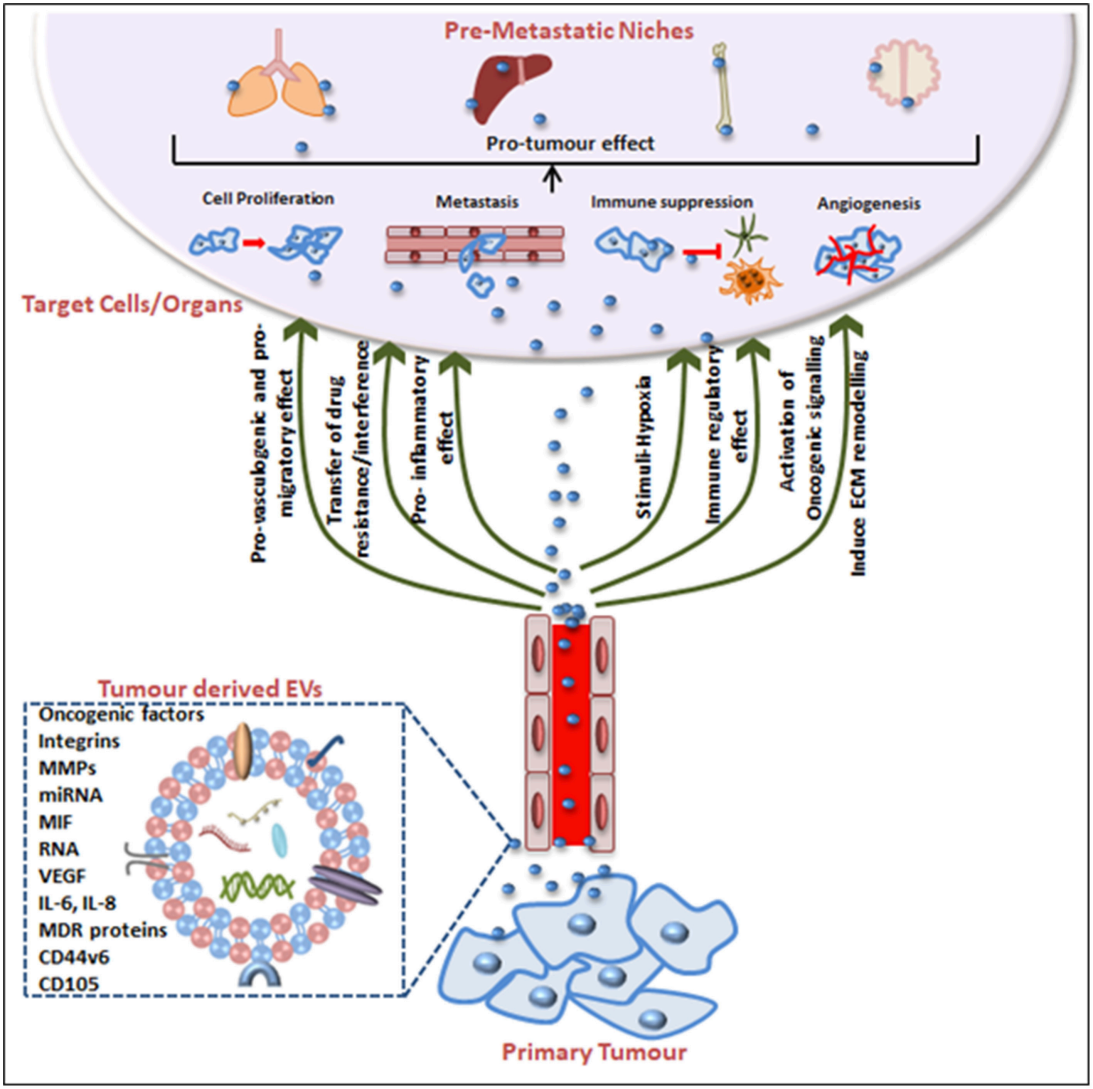
Tumor-derived EVs in pre-metastatic niche (PMN) formation and tumorigenesis. Tumor-derived EVs express surface and cytosolic molecules originating from the primary tumor and are carried to recipient cell/organs via the circulation. The EV surface molecules and cargo confer pro-angiogenic, pro-migratory, pro-inflammatory effects, and chemotherapeutic drug interfering or immune-regulatory effects. EV movement to target organs is generally organotropic and determined by the inherent tumor cell and EV cargos. Finally, EVs contribute to the pre-conditioning of the target site via inducing extracellular matrix remodeling, changes in blood and lymphatic vessels barrier integrity, transfer of immune inhibitory or activating factors and transfer of oncogenic factors. Together these mechanisms explain EV contributions to cancer progression and the impact on cancer treatment efficacy including treatment failures.

Tumorigenesis was historically attributed to genetic and epigenetic alterations in the genome of an organism but there is compelling evidence that EVs are integral to cancer cell communication and cancer progression. This is essentially due to EV contents because when produced from tumor cells EVs are a reservoir of cancer-associated molecules. Not only can EVs aide tumor cell survival (8, 175, 176) but certain cargoes educate or condition the recipient cell toward a tumor-promoting phenotype to facilitate the establishment of a pre-metastatic niche, thus promoting metastasis (155, 174). In fact, EVs can act as abettors of transformed cells, promoting their proliferation, propagation, even their chemotherapeutic drug resistance phenotype, their capacity for increased angiogenesis and stromal remodeling, and even in the evasion of immune detection (Figure 2).

### Direct Oncogenic Phenotypes

EVs can exert a cargo-dependent oncogenic response in recipient cells. For example, glioma cells display EGF-RvIII, the oncogenic form of the EGF receptor, which induces mitogen-activated protein kinase (MAPK) and Akt pathways promoting anchorage independent growth and survival (19). When compared with EV-donor cells deficient in EGF-RvIII expression, EGF-RvIII-expressing EVs display EGF-R-dependent responses, i.e., AKT and ERK signaling and oncogenic phenotype in EGF-RvIII-null recipient cells (19). Moreover, EGF-RvIII was demonstrated to subsequently present in the EV-interacting recipient cells (19). In another example, apoptotic body type EVs derived from H-ras^V12^ and c-myc oncogene transfected cells conferred tumorigenicity *in vivo* (in mice) via a mechanism involving oncogene transfer into recipient cells (177). The EV-donated DNA oncogene cargo is likely present episomally (rather than inserted in *cis*), but nevertheless transferred DNA can have oncogenic potential via DNA-encoding oncogenes or the oncoproteins themselves, in some respects resembling certain instances of non-integrating viral transformation (178).

Other pro-tumor EV effects include the role of the pro-coagulant transmembrane tissue factor molecule that modulates angiogenesis as well as metastasis in cancer [reviewed in (179)] and tissue factor-bearing MVs derived from colorectal carcinoma cells can contribute to a K-ras dependent cancer progression (180). These tumor EVs can be transferred between dissimilar populations of cancer cells, meaning EVs can potentially enable the propagation of aggressive/oncogenic phenotype between various sub-populations of cancer cells present within heterogeneous tumors (181). Tissue factor-bearing EVs also contribute to cancer-induced thrombosis (182). Thus, tissue factor-expressing MPs are mediators of tumor aggressiveness, a trigger of thrombogenesis and abnormal coagulation in cancer. These types of EV interactions are now being targeted in cancer-associated venous thromboembolism therapies (183, 184).

As alluded to above, tumor-derived EVs can cause the activation of specific pathways that support tumor growth and survival. Tumor proliferation can be promoted by gastric cancer-derived exosomes through the activation of the phosphoinositide 3-kinase (PI3K), Akt, and mitogen-activated protein kinase/extracellular-regulated protein kinase (MAPK/ERK) pathways (185). Cancer cell line-derived exosomes can activate the mitogen-activated protein kinase Ras-Raf-MEK-ERK pathway in monocytes, through the transport of receptor tyrosine kinases EGFR and Her2, and promote the survival of tumor-associated monocytes (186). This mechanism of cancer progression alters monocyte survival prior to the formation of tumor-associated macrophages and occurs directly within the tumor microenvironment (186). Theoretically, this mechanism would also have the capacity to action distally e.g., via circulating monocytes.

### Pro- and Anti-inflammatory Effects

Malignant cells modulate the tumor microenvironment via both their pro- or anti-inflammatory effects. The immunomodulatory effects of EVs are particularly well studied in macrophages due to their high propensity for endocytosis and the abundance and accessibility of their precursors - blood monocytes. Melanoma-derived exosomes have been shown to be capable of inducing a pro-inflammatory recipient cell effect by altering the cytokine and chemokine profiles of the target macrophage cells (187). Similarly, breast cancer-derived exosomes can induce increases in mRNAs of pro-inflammatory cytokines such as interleukin (IL)-6, tumor necrosis factor (TNF), granulocyte colony stimulating factor (G-CSF) and chemokine CCL2, through a Toll-like receptor (TLR)-2 stimulation and NF-κB in macrophages (188). In a separate study, NF-κB-dependent expression of IL-6 and TNF was present in gastric cancer-derived exosome cultured macrophages (189). We have also recently demonstrated that MPs secreted from both drug-resistant and drug-sensitive breast cancer cells polarize macrophages toward a pro-inflammatory state, producing increased IL-6 and TNF, and we hypothesize that this contributes to the establishment of a pre-metastatic niche (PMN) especially at secondary tumor sites (176). Furthermore, lung tumor-derived exosomes express surface HSP70 and activates a pro-inflammatory phenotype defined as elevated IL-6, IL-8, and monocyte chemoattractant MCP-1 (190), and TLR-dependent NF-κB activation (190).

There are also reports of an anti-inflammatory role of tumor cell-derived EVs. Melanoma and colorectal carcinoma cell line derived EVs can stimulate the release of transforming growth factor-β (TGFβ) from tolerogenic T-cells that promotes the generation of myeloid-derived suppressor cells (191). This suggests a mechanism that establishes an “immunosuppressive circuit” within the cancer, but with this knowledge comes the potential als for therapeutic modulation, e.g., by interfering with or blocking the interaction, i.e., to re-arm T-cell control of tumors (191). The challenge, however, resides in *how* to specifically inhibit *only* EV:TGFβ-producing tolerogenic T cells and not tumor-specific cytotoxic T lymphocytes, and or anti-tumor Natural Killer (NK) cells. Such interventions would need to be cytokine-specific. Other anti-inflammatory mechanisms of EVs includes gastric carcinoma cell-derived MPs interacting with monocytes resulting the production of the immunosuppressive cytokine IL-10. This is directly immune suppressive and additionally acts indirectly to decrease pro-inflammatory cytokines GM-CSF and TNF (via feed-back loops). Hence, EVs skew the tumor cytokine milieu to either an anti- or pro-inflammatory signature (192). Interestingly, EVs have been artificially packaged to deliver anti-inflammatory agent curcumin, i.e. to be delivered to distant site for the treatment of inflammatory conditions (193); curcumin has potent anti-inflammatory properties such as being capable of inhibiting the production of TNF (194). In fact the idea and potential potency of curcumin as an anti-cancer agent has led to the generation of synthetic curcumin analogs and these are being investigated in cancer (195, 196). Of note, however, curcumin itself appears to be capable of altering the EV cargo e.g., for enrichment of miRNAs with anti-cancer properties (197).

### Drug Interference and Resistance

Tumor-derived EVs can act as direct mediators of cancer resistance to chemotherapy, via a multimodal process. Horizontal transmission of drug resistance occurs through transfer of drug-resistance conferring molecules: the ATP-binding cassette (ABC) transporter p-glycoprotein (MDR1/ABCB1) (58, 139, 151), multi-drug resistance associated protein-1 (MRP1/ABCC1) (198), the multidrug resistance efflux transporter ABCG2 (199), the ABC transporter-3 (ABCA3) (200), the P-gp inducer- of Ca^2+^-permeable channel transient receptor potential channel-5 (TrpC5) (201), and the Her2 receptor protein ERBB2/EGFR2 (77). The significance is that EVs containing these cargoes confer a cancer drug resistance phenotype to recipient cells i.e., resistance to multiple tumoricidal drugs (58, 151). Moreover, we and others have demonstrated that modulation and transfer of certain miRNA, lncRNA and mRNA nucleic acids in EVs further regulate drug-resistance traits of tumor cells (139, 202, 203). EVs also function to attenuate the effectiveness of chemotherapeutics by sequestering the administered drug before it reaches the intended tumor. This occurs through the presence of the drug receptor or a drug-interacting protein within the EV, that essentially creates a sublethal concentration of the drug in the circulation of the patient (204). Others have shown that in the case of cisplatin-containing exosomes produced from melanoma cells this helps to generate a cisplatin-resistant melanoma phenotype (117). It is thus appears that cancer cells can directly accumulate drugs within the vesicular compartment and then eliminate the drug by EV shedding, and furthermore, that enhanced shedding correlates with increasing EV drug concentrations (205). In the case of Her2-expressing breast cancer, exosomes bind to trastuzumab antibody, resulting in drug resistance (77). Indeed, *in vitro* activation of Her2 (by heterodimerisation with EGFR or Her3) with ligands epidermal growth factor (EGF) and heregulin results in the increased production of exosomes (77). This suggests that cancer cells specially upregulate their EVs secretion in the presence of the drug. In the case of Her2-positive tumors, EV sequestration of trastuzumab also inhibits leucocyte activation and cytotoxic effector mechanisms for Her2-expressing tumors (206). Similar effects have been shown for B-cell lymphoma exosomes displaying CD20 with subsequent resistance to the anti-CD20 chimeric antibody rituximab (200).

## Effects on Immune Regulation Facilitating Tumor Progression

The production of EVs constitutes a mechanism of tumor-specific immune suppression. This is a complex situation involving multiple mechanisms. Firstly, the packaging of immunosuppressive soluble mediators such as TGFβ in EVs, can directly incapacitate cytotoxic anti-tumor T lymphocytes and NK cells (207, 208), or EV-derived IL-10 can activate myeloid-derived suppressor cells (MDSCs) (191, 209). So too, EVs carrying death-inducing cytotoxic cytokines TNF, TNF-related apoptosis-inducing ligand, TRAIL, and Fas ligand (FasL) induces apoptosis of tumor-specific T cells (210), and exosomal EVs block NKG2D to inhibit NK cell activation and NK tumoricidal activity (211–213). In other examples, MV surface TGF-β1 suppresses NK cell and T cell proliferation through adenosine production, and miRNAs such as miR-23a (214) and miR-4498 regulates CD83 expression (215)(CD83 is a dendritic cell activation/maturation molecule). Furthermore, tumor EVs are similar to the tumor cells themselves, in that they can contain tumor specific antigens such as melan-A and carcinoembryonic antigen (CEA) that are capable of suppressing tumor-specific responses (216). This has been dramatically demonstrated *in vivo* (in mice) using a model tumor antigen of ovalbumin (OVA)-in melanoma cell derived exosomes (217). Likewise, circulating, tumor-derived MHC Class-II bearing exosomes suppressed tumor-antigen specific responses in tumor-bearing mice (218).

EV-derived immunosuppression also occurs via tumor cell EVs promoting FoxP3^+^ T regulatory (Treg) cell proliferation or enhancing their suppressive capacities (219). Moreover, nasopharyngeal carcinoma-derived exosomes cause the conversion of naïve T-cells into the immunosuppressive Treg cells and promoted Treg recruitment via chemokine CCL20 (220). This is well demonstrated in an *in vivo* mouse study where the presence of metastatic breast cancer-derived exosomes leads to the establishment of a microenvironment augmenting cancer metastasis to the lungs and liver, in mice (221). Here, the continuous uptake of breast cancer exosomes in these organs led to the recruitment of immature myeloid cells, decreased numbers of T-cells and NK-cells, correlating with increased cancer progression and mortality (221). Similarly, glioma stem cell-derived exosomes suppresses T cells through monocytes (or myeloid-derived suppressor cells) leading to glioma immune-evasion (222). These are not considered uncommon findings or physiologically unlikely effects in humans, since the tumor-specific EV immunosuppressive effects can be demonstrated for EVs present in body fluids such as malignant effusions or the sera of cancer patients (191).

Finally, cancer-derived EVs can influence macrophage phenotype, converting M1 pro-inflammatory anti-cancer macrophages into M2 phenotype macrophages that better support tumor survival. For example, colorectal cancer-derived MVs regulated the differentiation of monocytes into regulatory M2 macrophages after prolonged contact (223), and glioblastoma-derived EVs induced a modified phenotype of monocytic cells to resemble myeloid-derived suppressor cells (MDCS) in the brain cancer patients (224). Finally, miRNA are potent regulators of immunity, and epithelial ovarian cell-derived exosomal miR-222, and pancreatic cancer-derived exosomes, have both been demonstrated to promote M2 macrophage phenotypes (225, 226). Conversely, over-expression of miR-155 and mI-125b-2 promotes an M1 macrophage phenotype (225). Thus, immunosuppressive tumor-derived EVs and their miRNA cargos modulate tumor immune responses and potently direct tumor immune evasion. A deeper investigation of EV-directed immune evasion mechanisms will be needed in order to harness EVs as effective adjunct cancer therapeutics.

## Pro-tumor Effect on Microenvironment

The interplay between the malignant cells and their neighboring healthy cells, stromal cells, endothelial cells, and the immune cells that girt and infiltrate a tumor, comprise the tumor microenvironment. Outcomes of such interactions are fundamental to the growth and spread of the tumor (227), and in this context EVs and EV-derived molecules critically modulate this microenvironment in a number of ways, including via an angiogenic effect, an invasive effect, and a metastatic effect.

### Angiogenic Effects

Tumors release angiogenic factors that support the development of new blood vessels to provide an adequate nutrient supply that facilitates tumor growth and metastasis. Tumor angiogenesis is stimulated by the proliferation of endothelial cells within the tumor (228) and also by the recruitment of precursor endothelial cells (from bone marrow) (229), where vessel endothelial cell pericytes aide blood vessel function and the capacity to generate new capillary structures. A role for EVs in microvasculature biology is evident within the tumor microenvironment where tumor-derived EV cargo molecules impact on angiogenic-modulating processes. For example, tumor-derived EVs carry pro-angiogenic factors such as IL-6 and VEGF stimulate endothelial cell proliferation and neo-vascularization within the growing tumor (71, 230), and EV nSMase2 stimulates endothelial cells and angiogenesis (231). Endothelial cell tube formation is stimulated by exosomes when the EVs are released from leukemic cells under hypoxic conditions, and develop within the central regions of growing necrotic tumors (146, 232). A deeper and more precise understanding of the molecular interactions between EV from cancer cells are currently being revealed: a recent mass spectrometry examination of EVs from Glioblastoma cell lines identified over 1000 proteins, including EGFRvIII, to exert angiogenic and tumor-invasive characteristics (233). Other EV angiogenesis promoting molecules include sphingomyelin (234), tetraspanin (235), and miRNAs miR-210, miR-9, miR-92a (232), viral oncoproteins (236), clotting factors and tissue factor (237), cytokines (238), and the oncogene Wnt5A (76) that promote tube formation by human vascular endothelial HUVEC cells (239). The list of clinically used and newly trialed chemotherapeutic agents for cancer, must therefore now consider not only the effect(s) of the drug on the tumor cells, but the effects of EVs on blood vessel endothelium physiology. Blood vessel permeability is particularly important in the setting of brain cancer, where increased intracranial blood pressures is an important clinical presentation, requiring clinical management through the co-delivery of anti-hypertensive agents. It is not surprising therefore that exosomes are also of interest in a number of related settings in neurology including cerebral ischemia where vascular function is integral to the pathology (240).

### Invasive Effects

The process of tumor cell invasion is assisted by matrix-degrading proteases and because EVs act as a constant inter-cellular communication system between the tumor and the stroma, EVs play an important role facilitating tumor invasiveness. Tumor-derived EVs promote extracellular matrix degradation, thereby assisting in tumor tissue infiltration. Such molecules include matrix metalloproteinases (MMP)-2, MMP-9, and MT1-MMP (241), their zymogens urokinase-type plasminogen activator (uPA) (242) and extracellular matrix metalloproteinase inducer EMMPRIN (89). Interestingly, malignant ovarian ascites samples from patients with stage-I to -IV ovarian cancer contain proteases MMP-2-, MMP-9-, and uPA- loaded EVs with highly invasive properties (61). In fact, there is a correlation between proteolytic and *in vitro* invasive capacity of EVs from breast cancer cell lines (242). Moreover, we have shown that breast cancer-derived MP cargo modulates miRNA-503 and proline-rich tyrosine kinase-2, leading to an ennhanced tumor migratory and invasive capacity (243), and it is well known that lung tumor derived-MVs modulate stromal fibroblasts and endothelial cells to promote tumor growth *in vitro* (86, 244). Indeed, tumor exosomes exert both stromal inducing pro-angiogenic effects as well as pro-invasive effects (245, 246) that aides tumor growth *in vivo*.

### Signaling Tumor Growth and Metastatic Effects

Metastasis involves the dissemination of cancer cells to adjacent or distant tissue sites. Tumor-derived EVs can deliver autocrine, paracrine, endocrine signals facilitating tumor growth and metastasis. Gastric cancer-derived exosomes stimulate the proliferation of other recipient gastric cancer cells, at least in part, through the activation of the PI3K/Akt and MAPK/ERK kinase pathways (185), and exosomes mediate the disposal of a tumor suppressor miRNA miR-23b that aids in the dissemination of cancer cell metastasis (247). Hypoxia-inducible factors and small GTPase Rab22A that mediate microvesicle biogenesis also facilitate breast cancer invasion and metastasis (248). So, too, overexpression of the ERBB2/Her2 oncogene in breast cancer-derived EV alters the vesicular contents toward a malignant phenotype (150). There are several examples of this phenomenon from *in vivo* models of cancer. Pancreatic carcinoma cell derived exosomes carrying CD44v6 promote tumor growth and metastasis in lymph nodes and lungs of rats (249). One of the main mechanisms for metastasis promotion appears to involve EV signaling in the tumor-surrounding stroma i.e., in the tumor microenvironment, with or without a role for transforming viruses. For example, gamma herpes virus Epstein-Barr virus infected cells secretion EVs containing integrins, actin, interferons and NF-κB that directly modulate the tumor microenvironment (236). In other *in vivo* examples, miRNAs such as miR-181c and miR-105 EV cargos of breast cancer cell-derived exosomes induce, or facilitate, metastasis to the brain by modulation blood-brain-barrier function (250, 251). Furthermore cancer-derived exosomes that promote lung metastasis use mechanism(s) involving the activation of TLR-signaling by the MyD88 adaptor protein, producing IL-6 and TNF-mediated activation of MDSCs (252). In yet a final example, lung cancer MVs promote metastasis via their expression of proteolytic matrix metalloprotease-9 (MMP-9) from fibroblasts (86) and through a fibronectin-integrin based pathway (253). Hence, it is generally accepted that EVs can enhance the creation circumstances that pre-conditions or educates the secondary metastatic site [for another recent review see (254)].

### Tumor-Secreted Effectors of Cross-Boundary Communication Leading to Organotropism and Pre-metastatic Niche Formation

EVs can influence remote organs and distal sites that are otherwise protected by physiological barriers. The creation of a pre-metastatic niche, i.e., a tumor supportive pre-conditioned environment, generally involves bone marrow-derived immunosuppressive cells - and a complex interplay between tumor cell determinants such as tumor-derived factors, and colonization pathways, and changes to metastatic site cells. In certain circumstances tumor-derived EVs can dictate the organ-specific migration of tumors (255–257), or, pre-metastatic niche creating EVs may originate from non-transformed healthy cells (258). It is generally thought that organ- or tissue- specific metastatic preference is due to endogenous niche components within the target organ (259–261). The role of EVs in organ-specific metastasis has been demonstrated by i.v. (tail vein) melanoma-derived exosomes that preferentially migrate to common melanoma metastatic sites of lung, spleen, bone marrow, and liver (155). Fusion of integrin-expressing exosomes (derived from lung-, liver-, and brain-tropic cancer cells) with organ-specific inhabitant cells results in Src-phosphorylation and pro-inflammatory *S100* gene expression (174). In this example, the specificity follows the general rules of integrin-homing, where the exosomal integrins α6β1 and α6β4 determines metastasis to the lung, while integrin αvβ6 promotes liver metastasis (174).

Metalloproteases are important in in cancer metastasis and tumor derived-EVs modulate the expressions of MMP-2, MMP-9, and VEGF-A directly at the metastatic site (241, 262, 263). MMPS can also act on blood (or lymphatic) vessels that aiding tumor cell recruitment and tissue matrix remodeling (264). There are also reports of pancreatic cancer exosome-mediated transfer of macrophage migration inhibitory factor (MIF) to liver Kupffer cells, subsequent to increasing TGF-β and fibronectin production, thus aiding tissue remodeling and future tumor metastasis (265). In breast cancer, MV-mediated transfer of miR-122 and inhibition of pyruvate kinase prevents glucose uptake and favors brain metastasis (266). Other examples of EV cargos that influence metastasis are miR-200 (267), tyrosine-kinase receptor expression and silenced Rab27A (155), and mesenchymal stem cell marker CD105- containing MVs (262); these factors all function to prime distant organs for tumor migration.

Essentially the *ex vivo* isolated tumor EVs have a pro-tumor effect by inducing modifications in remote organs, followed by the relocation of metastatic tumor cells. Thus there is a need to design strategies that manipulate pre-metastatic niche and thereby to prevent seeding and metastasis *in vivo* [for review see (268)]. Recent exciting advancements in this area highlight the capacity for oncomaterials that modulate tumor cell behavior at the metastatic engraftment site. Several oncomaterials, both natural products such as bone fragments, silk, collagen, lung/liver matrix, as well as synthetic derivatives including poly-L-lactic acid, hydroxyapatite, polyacrylamide, are already approved by the US Federal Drug Administration for their application to humans for tissue bio-engineering applications (268). This is a particularly exciting area of EV research and translational medicine for cancer patients, as it offers the future potential to prevent tumor metastasis.

## Tumor-Derived Extracellular Vesicles: Cancer's Friends or Foe?

One application of tumor-derived EVs is that they can constitute novel tumor-specific diagnostic targets, where their contents are diagnostic targets that indicate the presence (or absence) of a tumor. The EVs themselves, or the EV cargo molecules, can be used to track cancer risk, cancer presence, and as marker of treatment outcomes—cancer cure or relapse. In certain circumstances it would be highly beneficial to selectively diminish or deplete tumor-specific EVs in circulation, without affecting non-tumor EVs that have other physiologically important functions. A technology currently in development is capable of removing soluble factors and exosomes from the peripheral blood circulation (269) and is marketed under the term ADAPT™ (adaptive dialysis-like affinity platform technology). It is capable of removing soluble factors and exosomes from the peripheral blood circulation (269). For example, Her2osome™ therapy diminishes tumor-derived Her2-containing exosomes that functional inhibit Her2-binding drugs. This is an impressive example of the potential of this approach, however, there are many other exciting opportunities to selectively target EVs, or their contents, in acute or chronic disease—not just in cancer patients.

On the other hand, the production and presence of tumor-derived EVs can have undesirable effects for the host organism, although, the deleterious effects can also be modulated for better treatment outcomes for cancer patients. Thus, there can be benefits to developing and using compounds that inhibit or interfere with tumor cell EV biogenesis, release and recipient cell uptake. These include reagents that block phosphatidyl serine, surface heparan sulfate, proteoglycans, ICAM1 interactions with their receptors i.e., molecules that are critical for EV engulfment (172). For example, the blocking EV phosphatidyl serine by diannexin suppresses the growth of human glioma in murine xenografts (270, 271). In another example, targeting of exosomal FasL (via FasL-specific monoclonal antibodies) can reduce melanoma tumor growth (272). There are actually many such reagents that are being investigated by biotechnology and pharmaceutical companies worldwide; many are currently being assessed in human clinical trials (Table 2).

### EVs as Disease Biomarkers

EV cargoes are stable and have a long half-life to their encapsulation within the vesicular membrane. Even the EV themselves are relatively stable in bodily fluids, and they withstand long-term *in vitro* storage conditions including several freeze-thaw cycles, making them excellent biomarkers of disease (273). Indeed, the EV stable biomarker cargo, be it protein, lipid, nucleic acid, can include molecular entities that relatively short-lived or highly labile in the cytoplasm of donor cells. In cancer these molecules can be reflective of both the tumors presence and also of cancer staging. For example, EVs in blood and urine of prostate cancer patients contain unique prostate-cancer specific contents that are biomarkers of prostate cancer (274, 275) but even where the diagnosis *per se* does not necessarily equate to a need for treatment (as in prostate cancer), markers of cancer metastasis are particularly valuable to cancer clinicians. Periostin, is an example of an EV metastatic cancer bio-marker. Periostin is concentrated at high levels in EVs derived from metastatic cells *in vitro* and abundantly present in plasma exosomes in breast cancer patients with lymph node metastasis (276). Periostin is also present in muscle-invasive bladder cancers, and urinary EV from patients is a prognostic biomarker of muscle invasiveness (277). Moreover, *in vitro* periostin knock-down experiments demonstrate reduced tumor migration and invasion, in model systems (277), thus EV periostin maybe also functionally important in tumor progression. EVs are proving to be valuable diagnostic biomarker in pancreatic cancer; flow cytometry coupled with mass spectrometry analysis of exosome glypican-1 can distinguish benign disease from early and late stage cancer (278). The challenge now is how to implement these technologies into routine laboratory testing.

Where the important EV cargo is nucleic acids quantitative PCR and/or next-generation sequencing is easily possible. To date, ssDNA, dsDNA and retrotransposon content of EVs have been profiled by PCR (279). However, the presence of 8 miRNAs of diagnostic importance in ovarian cancer, can be specifically amplified and detected by reverse-transcription PCR, even in as yet asymptomatic patients (56). Other similar examples continue to be reported: human lncRNAs profiling of prostate cancer cell lines and their exosomes has identified several cancer-specific RNAs in exosomes (280). Independent validation of these markers is required to progress the development of diagnostic assays for future routine use in pathology laboratories.

EV DNA nucleic acid is not transcriptionally competent since whilst in the EV as there is no RNA-polymerase-II transcriptional complex present there. The EV DNA is simply present and potentially being transferred to a recipient cell. Once transferred, this DNA may have potential for nuclear import and at some point, could theoretically regain transcriptional potential, even if only present episomally. Similarly, EV mRNAs are not translated whilst within EV, as cellular ribosomes are not present within EVs. There is evidence, however, that once transferred, mRNA can potentially be expressed in the recipient cell (281). In contrast, however, we have shown that EV miRNA has the potential to be carried and *produced* within the EV, because the miRNA biogenesis molecules are also present within the EV (139). The significance of EV RNA transfer, including non-coding RNAs, is clearly that it may bring a regulatory RNA into the recipient cell i.e. that is otherwise not expressed endogenously within the recipient cell. EV transferred nucleic acids can therefore change the protein expression profiles of recipient cells.

### EVs as Therapeutics for Targeted Cancer Drug Delivery and More

That EVs strongly preserve diverse bioactive cargos immediately places them as high-utility delivery vehicles, targeted to particularly recipient cells. Arguably one of the most easily achievable applications of therapeutic EVs is that they can be manufactured to deliver cancer chemotherapeutic drugs, such as doxorubicin, paclitaxel, methotrexate or cisplatin, etc., singly or in combination. This can be achieved by an approach as simple as preparing the EVs from cells that are cultured in the desired chemotherapy drug, or combination of such drugs (282) and early indications are that this targeted delivery may reduce toxicity-related adverse effects (283–285). At this stage there would seem to be a broad range of molecules that can be manufactured for delivery via therapy EV. Examples to date include anti-cancer agents such as the angiogenesis and cancer growth inhibitor withaferin A (286), or the plant-based triterpenoid-celastrol (287). In both of these cases, delivery of the therapy EV resulted in a stronger anti-tumor effect in a human lung tumor mouse xenograft model compared to the free drug alone, presumably due to better tumor-specific drug targeting (286, 287). In other examples, curcumin, has been loaded into EL-4 lymphoma cell-derived exosomes and delivered to activated myeloid cells, for protection against LPS-induced inflammation in mice (288), and intra-nasal exosomal delivery of curcumin, or a signal transducer and activator of transcription-3 (STAT3) inhibitor JSI124, has been shown to cross the blood-brain-barrier and suppress GL26 brain tumors in a syngeneic C57BL mouse model of brain cancer (193). Others have prepared EVs directly from plants, and a clinical trial of plant-derived EVs is already underway (see Table 2). While these results are exciting developments, especially for patients with poor-prognosis tumors like glioblastoma, much more information is required to better understand the immunological ramifications of EV therapy, especially in the central nervous system. Although it might be necessary to employ one or more monocyte/macrophage phagocytosis blocking strategies, the small size of EVs has been demonstrated to bypass the blood-brain-barrier endothelium for CNS tissue delivery (289) (for review see (240).

EVs are being pursued as intercellular vectors for RNA-based therapy (both miRNAs and siRNAs), with efficacy documented in animal models of disease. For example, *in vivo* suppression of breast cancer and prostate cancer has been achieved through exosome delivery of the tumor suppressor miRNAs miR-let-7a and miR-143 in mice (290, 291). Other examples include exosome transfer of miR-146b to glioma cells to inhibit glioma cell growth (292), or exosomes released synthetic miR-143 to osteosarcoma cells to limit migration (293). In these circumstances the RNA cargo need not be a naturally occurring miRNA within the recipient cell. Moreover, siRNA-loaded EVs can specifically deliver anti-tumor effects (294). siRNAs targeting RAD51 (295), c-Myc (296), and PLK-1 (297) inhibit the proliferation of breast cancer, lymphoma, and bladder cancer cells, and “suicide gene” mRNA MVs have efficacy for treatment of pre-established nerve sheath tumors (schwannoma) in an orthotopic mouse model (298).

Although there is an expanding list of these proof-of-principle type experiments, there are currently also a list of challenges that remain, especially in the era of individualized or person-specific targeting of cancer. There is also the issue of what happens in situations of cancer recurrence, and whether chemotherapy-loaded EVs can be re-administered or have immunogenicity in their own right. This is especially of concern, if or when EVs are phagocytosed by macrophages and tissue-resident dendritic cells as this could work to raise antigenicity of the tumor-specific EV-delivered molecule, or the (modified) EVs themselves. If the therapy EVs were autologous (and patient specific) this would help address the potential for immunogenicity. Interestingly, the present thinking appears not to consider EV HLA-expression as being a significant factor, but we have evidence of some degree of immune cell activation by non-HLA-matched EVs (Jaiswal and Sedger, unpublished). A deeper understanding of the benefits and/or limitations of EV immunogenicity may prove to be beneficial to the success of therapy EVs, and it may prove to be beneficial to examine this more deeply in current cancer clinical trials and more human data is desperately needed.

The issue of immunogenicity is a double-edged sword. The potential for therapy EVs to enhance immunogenicity is particularly useful given that EVs are well known to carry tumor-associated antigens (TAAs). Therapy EVs have a documented capacity to elicit TAA-specific anti-tumor immune responses, which improves cancer survival in primary and metastatic mouse tumor models (299). Furthermore, the tumor antigen human mucin-1 (hMUC1), delivered via therapy EVs, has been utilized in dendritic cell-based immunotherapy (300). Hence, TAAs can elicit a strong anti-tumor immune response when fused with the C1C2 domain of lactadherin that binds to the phosphatidyl serine on the EV surface (301, 302). Significantly, the TAA laden EVs can result in “cross-presentation” of tumor-specific antigens in recipient cells (301, 302) in a manner that closely resembles that of apoptotic bodies. This means that an endogenous pathway of antigen processing and presentation can be affected by EVs for tumor cytotoxic T cell recognition. The enhanced immunogenic potential of this approach has been demonstrated for the TAAs carcinoembryonic antigen (CEA) and Her2 (303, 304). Also, tumor-derived MPs inducing the type-I interferons (through the cGAS/STING pathway) resulted in increased dendritic cell maturation, and in enhanced tumor specific T-cell tumor lysis (305). Hence there are many ways in which tumor MPs exert adjuvant effects. Indeed, there is already exciting evidence that exosomes isolated from the ascites fluid of cancer patients can function as a personalized vaccine for cancer (43) and phase-I clinical trials have been performed to evaluate autologous dendritic cell-derived cancer exosome therapy for lung and melanoma cancers (306). This is an active area in clinical cancer medicine; a list of current human clinical trials utilizing EVs in cancer is provided in Table 2.

## Current Challenges in the Use of EVs in Oncology

As with any new or emerging biotechnology there are technical issues surrounding its use in the clinic. For EVs these include difficulties in standardization of EV isolation techniques and EV detection methodologies, as well as issues related to the GMP production of therapy EVs.

### Challenges in Assessing Biomarker EVs of Clinical Relevance

The potential uses of EVs as biomarkers is exciting but caution is needed as there are many different methods of isolating, detecting and using EVs in cancer detection and treatment (307, 308). This immediately highlights the need for methodological standardization for EV assessment and its introduction into routine pathology laboratories, in order to achieve reproducibility and clinician confidence in the data. Secondly, the assessment methodologies require determination of the level of sensitivity, and the false discovery rate, as these are important for accurate clinical use (309, 310). Detection of proteins can be problematic unless there are high quality monoclonal antibodies for affinity methods, whereas nucleic acid is easily quantified with high degree of accuracy. Furthermore, EV isolation methods and detection efficiencies are integrally linked, because the EV isolation method can impact on EV phenotype and cargo detection capabilities (311). This is partly due to inherent heterogeneity of the EVs in the starting biological sample, and the remaining sample heterogeneity after EV purification [see Table 3 and 4 in (310)]. Traditionally, centrifugation-based methods have been used for EV isolation in research settings. However, the type of centrifuge (with fixed or swinging bucket rotors), the centrifugation matrix/medium, centrifugation speed and time, influences the quality of the EV isolation [thoroughly reviewed in (312)]. The viscosity of the starting sample (and hence both the sample and centrifugation temperature) also impacts on centrifugal methods (313). Step-wise density centrifugation and ultrafiltration offers some improvements (314), including the capacity to add artificial standards, but density gradient centrifugation is time-consuming and has limitations in laboratory scale up (312). Other methods for EV isolation include size-exclusion chromatography, or polymer-based precipitation especially by substances such as polyethylene glycol and sodium chloride or commercial products. The number of methods highlight the difficulties in standardization for EV preparation, and several groups are now reporting optimized isolation methodologies based on the biological sample type and the intended EV use (315), including EV isolation for miRNA or protein profiling (316, 317). Furthermore, given the known presence of specific molecules in certain EVs, immuno-affinity-based methods are also now common, with both “positive” or “negative” affinity selection methods available, offering high selectiivity and high purity (318, 319). The advantage of the immune-affinity approach is that EV particle size heterogeneity is no longer an issue; any particle with the antibody-targeted molecule can be purified. One disadvantage, however, is that not all EVs produced from a cancer may express the designated isolation marker(s), such that certain useful EVs will be lost by antigen-specific immune-affinity based isolation procedures [reviewed in (312)].

Additional innovative methods of EV detection and characterization include various microfluidic (e.g., “lab-on-chip”) approaches that allow for efficient molecular capture and quantification from relatively small volumes of circulating exosomes (320). Indeed, EVs can now be detected even as little as one microliter of patient plasma, which is achieved by coupling antibody-conjugated gold nanospheres and chip-based microfluidic technology (321). In this regard innovative nano-plasmonic sensors (322), or tunable-resistive pulse sensing have been developed (323) and a nanoplasmon-enhanced scattering assay has been successfully applied to staging tumor progression and therapy responsiveness (324). However, despite these successes, there is still the significant problem that few detection assays incorporate biological reference materials - required for inter-laboratory standardization. It is currently possible to produce an EV reference material from synthetic sources, such as liposomes; these can be relatively easily prepared to a desired size by either ultrasonication or polycarbonate filtration (310). EV reference material can also be produced from naturally occurring biological sources such as mammalian erythrocytes (310). Various biological reference materials are currently being investigated for diagnostic assay purposes [for a recent review on this topic see (310)]. Hence, despite the available technologies to detect EVs, there is a clear need to establish assay performance quality control and “best-practice” for clinical laboratory EV detection and quantitation.

### Challenges in Using Therapeutic EVs

One of the earliest applications of EVs in cancer was the use of dendritic cell secreted exosomes. The idea is that cancer-peptide-loaded major histocompatibility together with co-stimulatory molecules born by exosomes confer EVs with the properties required for use as a cancer vaccine (299). Their size, structure, cargo, and mode of recipient cell uptake, make EVs prime vehicles to induce antigen cross-presentation to enhance tumor antigenicity (55). Pre-clinical mouse tumor studies showed efficacy of dendritic cell EVs, and now a number of human clinical trials are underway [(308, 325, 326) see Table 2]. Nevertheless, one of the limitations of using therapy EVs is obtaining sufficient quantities i.e. of tissue- or disease- specific, and functionally intact, preparations of EVs from body fluids. Even the relative abundance of urine as an EV bio-fluid source has a limit, and both patient and cell culture-derived EVs need to be “defined,” measured, and determined with high assurance for safe use in patients. Also, the use of EVs as cancer vaccines, requires *a priori* the existence of true immunogenic “cancer antigens” but these are usually not well defined, if defined at all. The “antigen” might constitute a re-activated endogenous retroviral antigen or cancer-specific neo-antigen. Whether cancer-EVs are pan-specific (i.e., for a selected cancer type) or whether they must be individually identified and isolated and used autologously, i.e., each individual patient, currently remains unclear. Furthermore, the optimal mode of EV delivery is also unclear in terms of achieving optimal clinical outcomes; delivery might be via i.v. infusion (single or repeated dose), or via an implanted medical device—for sustained release. Indeed, optimal delivery will possibly depend on the setting in which the EV vaccine is to be applied—an infectious disease, a certain cancer type, a primary cancer diagnosis or a cancer re-occurrence. On the other hand, the potential clinical use of tumor EVs carries with it potential dangers because of certain cargoes. For example, cancer cell derived EV can contain p-glycoprotein that confers resistance to chemotherapy drugs (5, 151), and certain EV cargo molecules enhance a tumor's metastatic characteristics (327, 328) (as discussed earlier). Clearly, therapy EVs require careful evaluation *prior* to their use in cancer clinical trials. Finally, in order to address the limited supply, and the various safety issues, plants and various food substances, including milk, have been investigated as a source of therapy EVs (286, 329, 330). An advantage of this source of EVs is the ease of scalability for clinical production. However, there is also the capacity to produce engineered EVs for specific purposes [recently reviewed in (331)], e.g., cancer chemotherapeutic agents such as paclitaxel and taxol can be easily incorporated into EVs (332). Finally, EV engineering has demonstrated the possibility to load the EV with a biosensor agent for sensitive *in vivo* monitoring to track EV bio-distribution (333).

## Conclusions

A vast repertoire of proteins and nucleic acid molecules are present within EVs and this reflects the broad potential of EV as therapeutic and diagnostic (and/or prognostic) agents. With the value of more precise analysis technologies, our understanding of the biological interactions of EVs for intercellular communication is rapidly expanding. This new knowledge has high relevance and high value for the use of EVs in cancer, including for innovative, even patient-specific “designer” cancer treatments. These developments offer the promise of more specific, less toxic, high-efficacy cancer treatments, especially in this era of personalized medicine.

## Author Contributions

RJ wrote the first draft of the manuscript. RJ and LS both contributed to manuscript revisions, read and approved the final version.

### Conflict of Interest Statement

The authors declare that the research was conducted in the absence of any commercial or financial relationships that could be construed as a potential conflict of interest.

## Abbreviations

EV: extracellular vesicles
LO: large oncosome
MP: microparticle
MV: microvesicle.
